# Do Intellectually Gifted Children Have Better Planning Skills?

**DOI:** 10.3390/jintelligence13050054

**Published:** 2025-05-13

**Authors:** Li Cheng, Xiaohe Xie, Shiting Yang, Linjie Xiao, Xiaoyu Chen, Yun Nan, Dong Qi, Jagannath P. Das, George K. Georgiou

**Affiliations:** 1 Faculty of Education, Beijing Normal University, Beijing 100875, China; 202431010044@mail.bnu.edu.cn (L.X.);; 2 Educational and Developmental Research Center of Children’s Creativity, Beijing Normal University, Beijing 100875, China; 3 Faculty of Psychology, Beijing Normal University, Beijing 100875, China; 4 State Key Laboratory of Cognitive Neuroscience and Learning & IDG/McGovern Institute for Brain Research, Beijing Normal University, Beijing 100875, Chinadongqi@bnu.edu.cn (D.Q.); 5 Department of Educational Psychology, University of Alberta, Edmonton, Alberta T6G 2G5, Canada; j.p.dasedmonton@gmail.com (J.P.D.); georgiou@ualberta.ca (G.K.G.)

**Keywords:** China, executive functioning, giftedness, intelligence, PASS theory, planning

## Abstract

The present study aimed to examine whether intellectually gifted children had better planning skills than their chronological-age controls and what processing skills may explain these differences. A total of 35 intellectually gifted Chinese children (25 boys and 10 girls; *M*_age_ = 12.77 years) and 39 chronological-age controls (27 boys and 12 girls; *M*_age_ = 12.89 years) participated in this study. They were assessed on three measures of operational planning (Planned Codes, Planned Connections, and Planned Search), on a measure of action planning (Crack the Code), and on measures of processing speed, working memory, and attention. Results of analysis of variance (ANOVA) showed first that the two groups differed in Crack the Code (accuracy and first move time) and in Planned Connections. Whereas processing speed explained the group differences in Planned Connections, none of the processing skills were able to eliminate the group differences in Crack the Code. Taken together, these findings suggest that gifted children have better action planning, which allows them to perform better than controls in tasks that require complex problem solving and evaluation of different scenarios and solutions.

## 1. Introduction

Executive functioning (EF), broadly defined as a set of abilities that an individual uses for the purpose of achieving a goal (Diamond 2013), has been found to be a significant predictor of academic achievement in different languages (Clark et al. 2010; Lan et al. 2011; Viterbori et al. 2015). The pinnacle of EF is thought to be planning, defined as “any hierarchical process in the organism that can control the order in which a sequence of operations is to be performed” (Miller et al. 1960, p. 16). Planning processes allow individuals to organize their conscious activity once information has been coded so that plans of action can be made, performance is regulated, and self-correction is possible. To date, despite the fact that EF has been extensively studied (see Spiegel et al. 2021, for a meta-analysis on its association with academic achievement), planning has been missing from most EF studies (i.e., EF studies include mostly measures of working memory, shifting, and inhibition), particularly among studies with gifted children. In addition, the few studies that examined planning in gifted children (e.g., Bucaille et al. 2023; Georgiou et al. 2022; Rocha et al. 2020) have not differentiated between the different levels of planning (e.g., action planning, operational planning), and we do not know if gifted children perform better than controls across all levels. Thus, in this study, we sought to examine the role of planning in intellectually gifted children.

### 1.1. Cognitive Planning

Planning has generally been viewed as part of EF, and several studies have included measures of planning when investigating EF (e.g., Carlson et al. 2004; Cirino et al. 2018; Lehto et al. 2003; Nemati et al. 2017). In a recent study examining the factor structure of different EF measures, Laureys et al. (2022) found that planning (operationalized with an adaptation of the Tower of London task) was a separate factor of EF and it correlated 0.39 with working memory, 0.27 with inhibition, and 0.16 with shifting of attention.

Several theories have also integrated planning into their models. For example, in Shallice’s Supervisory Attentional System (SAS) model, planning was thought to be governed by the SAS, a prefrontal cortex-based system that, among other things, handles novel or complex tasks. Likewise, in Baddeley and Hitch’s (1974) working memory model, planning was thought to rely on the central executive, a component of working memory that coordinates attention, decision-making, and strategy formation. One of the theories that has reserved a special place for Planning is the Planning, Attention, Simultaneous and Successive (PASS) theory of intelligence (Das et al. 1994). Das and colleagues (e.g., Das et al. 1994; Naglieri and Das 1987; Naglieri and Otero 2018) considered planning as the first operational system in the PASS theory of intelligence that is responsible for controlling, organizing, and monitoring behavior.

The PASS processes have been operationalized by the Cognitive Assessment System (CAS) battery (see Naglieri and Das 1997; Naglieri et al. 2014) that entails 12 measures (three for each PASS process). According to Best et al. (2011), the three measures of Planning in CAS (i.e., Planned Connections, Planned Codes, and Planned Number Matching) can be used to operationalize “complex” EF, an argument that is in line with the view of planning as the pinnacle of EF (Cristofori et al. 2019). The factor structure of CAS has been confirmed in several previous studies in different languages (Naglieri and Das 1987; Naglieri et al. 1991; Nakayama et al. 2012; Natur 2014; Wang et al. 2010). In addition, following the premises of CAS, Das et al. (2020) published the Brain Based Intelligence Test (BBIT) battery that also includes measures of planning such as the Crack the Code, Planned Connections (called Trails in BBIT), Planned Patterns and Planned Search (called Visual Search in BBIT). Again, the factor structure of these measures has been confirmed (Das et al. 2020).

### 1.2. Planning in Intellectually Gifted Children

Although measures of planning have been used in several EF studies, there is still a paucity of research on planning in intellectually gifted children. To our knowledge, only a handful of studies have examined the role of planning in intellectually gifted children, and they provided mixed findings (Bucaille et al. 2023; Georgiou et al. 2022; Naglieri and Kaufman 2001; Montoya-Arenas et al. 2018; Rocha et al. 2020; Vogelaar et al. 2019). The initial study on this topic was conducted by Naglieri and Kaufman (2001), and it was a single-case study. Using CAS, Naglieri and Kaufman found that Donna had a standard score of 139 in Planning. On the basis of this, they concluded that while more traditional IQ tests would not identify a child like Donna as gifted, the CAS does measure an important dimension of her giftedness. In turn, working with a group of Grade 4 to 6 intellectually gifted children, Georgiou et al. (2022) reported that their mean standard score in Planning (using the CAS measures) was 110.3 (high average), and only 5% of these children had a relative strength in Planning. Interestingly, Georgiou et al. also found that 18% of these children had a relative weakness in Planning. Montoya-Arenas et al. (2018), Vogelaar et al. (2019), and Rocha et al. (2020) reported no significant differences between their groups in planning. Finally, Bucaille et al. (2023) reported significant differences between gifted children and controls only when they asked parents to provide information on their children’s planning skills and not when they asked teachers to fill out the same survey (BRIEF; Gioia et al. 2015). Clearly, more research is needed on this topic.

Notably, none of the forementioned studies have examined whether intellectually gifted children perform better than their controls across different levels of Planning. In fact, in their book on cognitive planning, Das and Misra (2015) argued that the measures of Planning in CAS might measure lower-level planning skills and suggested that one way to further distinguish between the Planning and Attention scales of CAS is to add higher-level planning tasks (e.g., Crack the Code; see Method for a description). If this is true, it may explain why Georgiou et al. (2022) failed to find significant differences between gifted and non-gifted individuals. It is possible that gifted children excel when the tasks require higher-level cognitive planning, and the CAS planning tasks do not adequately capture that.

Alexei Leontjev, a prominent figure in Soviet psychology and a student of Lev Vygotsky, was among the first researchers to propose that planning has different levels. His work on levels of planning provides a framework of understanding how individuals develop and refine their ability to plan and regulate goal-directed behavior. Leontjev (1978) proposed that planning has three levels (activity planning, action planning, and operational planning) within activity systems that help structure human actions and decision-making. At the level of activity, planning can be thought of as a method of realizing or achieving one’s general life goals and motives over an extended period of time. This level deals with the “why” of activities—why we engage in particular activities in the first place, based on broader social or personal values, cultural norms, and long-term goals. For example, a student plans their academic path (e.g., choosing a major) based on career aspirations and after taking into account the market needs. In turn, action planning is similar to problem solving, and it serves as a bridge between strategic, long-term goals and concrete, short-term actions. In this level, individuals determine the practical methods and actions they will use to achieve the strategic goals. This includes forming a mental representation of the problem, the constraints on planning, and the course of action. In other words, this level deals with “how” an activity can be executed effectively, given current resources and constraints. For example, the same student creates a weekly study timetable to prepare for exams. Finally, operational planning deals with the immediate, day-to-day execution of tasks and decisions. Because the goal or end result is often known, operational planning involves forming a representation of the problem, choosing the possible operations to be applied, and then executing these steps. In other words, this level deals with the specific steps that are needed to achieve a goal. For example, the same student highlights key terms in passages while studying. The Planning tasks in the Das–Naglieri CAS battery (Naglieri and Das 1997) are good examples of operational planning (see Parrila et al. 1996, for empirical evidence).

An issue that also remains unclear in the literature is what processes may explain the possible differences between gifted and non-gifted children in Planning. Several researchers have already shown that Planning, being part of EF, is interconnected to other measures of EF such as working memory and inhibition (e.g., Cirino et al. 2018; Laureys et al. 2022; Lehto et al. 2003), and therefore, performance in these processes may interfere with performance in planning. As planning requires retaining multiple steps and adjusting strategies based on new information, it should correlate significantly with working memory. Some studies have already documented that gifted children have better working memory than their controls (e.g., Asensio et al. 2023; Aubry et al. 2021). Likewise, because effective planning requires inhibiting off-task behaviors and staying focused on long-term goals, it should correlate significantly with measures of inhibition (Diamond 2013).

Researchers have also argued that the relationship between different EF components may be partly explained by their shared speed requirements (e.g., van der Sluis et al. 2007; see also Demetriou et al. 2013, 2014, for a discussion on the connection between speed and fluid intelligence). For example, measures of planning like the Tower of London require children to provide an answer within a specific time limit. Measures of inhibition, like the Color–Word Stroop, also require children to name stimuli as fast as they can. The speed requirement of both tasks may explain (at least partly) their relationship. Because most Planning tasks require children to perform a task as quickly as possible or within a certain time limit (see e.g., Planned Codes, Planned Connections in CAS) and gifted children have better speed of processing than their chronological-age controls, gifted children may perform better than their chronological-age controls not because of their superior planning skills but because they process information much faster than their controls (see Kranzler et al. 1994). Thus, in this study, we measured not only children’s planning but also their working memory, inhibition, and speed of processing.

### 1.3. The Present Study

The purpose of the present study was twofold: (a) to examine if intellectually gifted children perform better than controls in different levels of planning, and (b) to examine what processing skills (e.g., processing speed, working memory, and inhibition) may explain their superior performance (if any). We hypothesized that intellectually gifted children would perform better than their chronological-age controls in action planning but not in operational planning. We did not formulate any specific hypotheses regarding the processes that may explain these group differences. We acknowledge here that because activity planning deals with long-term goals and the actions that an individual can take to achieve these goals over an extended period of time (e.g., a month, a year), we could not measure it in this study.

## 2. Materials and Methods

### 2.1. Participants

A total of 35 intellectually gifted children (25 boys and 10 girls; *M*_age_ = 12.77 years, *SD* = 1.01) and 39 chronological-age controls (27 boys and 12 girls; *M*_age_ = 12.89 years, *SD* = 1.00) participated in this study. The participants were recruited from two public schools in an underdeveloped rural area (an underdeveloped area is defined as a region with a per capita income below 60% of the national average; Fan et al. 2020) in XXX (name removed for blind review). Based on the norms of the Test of Nonverbal Intelligence-Second Edition (TONI-2; Borland 2009) for underdeveloped rural areas in China (Cheng 2024), intellectually gifted were those children with scores at or above the 97th percentile (mean percentile = 98.09, *SD* = 0.89, range = 97–99), and typically-developing children were those with scores at or below the 88th percentile (mean percentile = 65.36, *SD* = 14.74, range = 42–88). None of the participants had any attentional, neurological, or learning difficulties. In addition, none of the intellectually gifted children were coded as twice exceptional. Written consent was obtained from children’s parents prior to testing, in accordance with the Declaration of Helsinki. This study was also approved by the Institutional Review Board of Beijing Normal University (ICBIR_A_0024_006).

### 2.2. Measures

#### 2.2.1. Nonverbal IQ

The Chinese version of TONI-2 (see Zhang et al. 2003) was administered to assess children’s nonverbal IQ. We chose this task over other measures of nonverbal IQ because this is the only one with norms for children in underdeveloped rural areas in China. TONI-2 measures nonverbal abstract and figural problem-solving abilities in individuals aged 5 to 85 years across eight areas: shape, position, direction, rotation, contiguity, shading, size, and movement. Children were presented with a visual configuration of different designs and were instructed to choose the best-fitting fragment among five options to complete the pattern. A participant’s score was the total number correct (max = 63). Cronbach’s alpha reliability in our sample was 0.96.

#### 2.2.2. Planning

Because this manuscript was prepared for the special issue on PASS processes in this journal, our choice of planning tasks was partly dictated by this reason. To operationalize action planning, we administered the Crack the Code, and to operationalize operational planning, we administered Planned Connections, Planned Codes, and Planned Search. Planned Connections and Planned Codes make up the planning factor in the Cognitive Assessment System-2 (CAS-2; Naglieri et al. 2014), and Planned Search was included as a third measure of planning in the initial studies on PASS (Das et al. 1994). All four measures of planning have been used in previous studies in Chinese (e.g., Cai et al. 2013, 2016; Wang et al. 2010, 2012a, 2012b; Wei et al. 2018) and their psychometric properties have been tested and validated (e.g., Deng et al. 2011; Wang et al. 2010).

**Crack the Code (CTC).** CTC is based on the popular Mastermind game and was developed in 1983 by J. P. Das (Das and Heemsbergen 1983; see Das and Misra 2015; Das et al. 2020, for the items). Since then, it has been used in several studies, including studies with same-age participants as in our study, showing good psychometric properties (Cai et al. 2016; Das and Georgiou 2016; Papadopoulos et al. 2005; Parrila et al. 1996). Participants were presented with two to four lines of information, each containing three to four colored chips in a specific order. A label to the right of each row indicated how many of the colored chips were correctly positioned. Participants were asked to determine the correct sequence of colored chips based on the limited information. A practice item with one line of information, including two colored chips, was provided before testing. The task consisted of nine items with increasing difficulty, and each item was given a three-minute time limit. The task was discontinued after two consecutive errors. If children failed the practice item twice, the test was discontinued. Two indices were recorded: first move time and accuracy. First move time was measured as the time (in seconds) taken to make the first move of a chip to an empty position on the answer grid. The total number of correct answers was also calculated. For children who were unable to start the formal test, their total number of correct answers was recorded as zero, and their first move time was recorded as a missing value. Cronbach’s alpha reliability in our sample was 0.88.

**Planned Connections.** The Planned Connections task was adopted from the Das–Naglieri Cognitive Assessment System-2 (Naglieri et al. 2014). Children were required to connect a series of boxes containing numbers or letters in the correct sequence as quickly as possible. The task included two sets of items: one featuring numbers (1–25) and the other featuring both numbers (1–13) and letters (A–M). For the number-only items (three trials), children were asked to connect the numbers in sequential order. For the number-and-letter items (four trials), children were instructed to connect the numbers to the letters in an alternating sequential order (e.g., 1–A–2–B–3–C, etc.). Before testing, examples of both item sets were provided as warm-ups. The time taken to complete each item was recorded (in seconds). The final score was the total time taken to complete all items. Cronbach’s alpha reliability in our sample was 0.84.

**Planned Codes.** The Planned Codes task was also adopted from the Das–Naglieri Cognitive Assessment System-2 (Naglieri et al. 2014). Children were asked to translate letters into specific codes and fill in the boxes as accurately and quickly as possible. The task consisted of four pages, each containing items arranged in a grid of eight columns by four rows, with each box marked with one of the letters A, B, C, or D. The code system, consisting of X’s and O’s (e.g., A = XO, B = OO), was displayed at the top of each page. Three different coding arrangements were applied in the task. Three samples were provided to ensure children understood the task. The time taken to complete each page (in seconds) and the number of correct items on each page were recorded and subsequently converted into ratio scores to determine the final scores. Cronbach’s alpha reliability in our sample was 0.84.

**Planned Search.** The Planned Search task was adapted by Das (1980) from the test of Teuber et al. (1949), and it was used as a measure of planning in the early studies on PASS processes (Cormier et al. 1990; Leong et al. 1985; Parrila et al. 1996). It is also now included in the Brain-Based Intelligence Test battery along with Planned Connections and Planned Patterns (Das et al. 2020). Children were instructed to match the target in the center box with the corresponding target in the visual field as quickly as possible. The target could be an object, a number, or a letter. The task consisted of 16 items, each comprising two searches. Before taking the test items, a sample item was provided to ensure children understood the instructions. During the task, children were given 90 s per item, and the time taken to complete each search was recorded (in seconds). The score was the total time required to complete all items. Cronbach’s alpha reliability in our sample was 0.80.

#### 2.2.3. Processing Speed

Children’s processing speed was measured by the Visual Matching task (Woodcock and Johnson 1989). In this task, children were required to circle two identical numbers within each six-number row as quickly as possible within a 3-minute limit. There were 59 rows arranged in order of increasing difficulty. One point was given for each correct answer.

#### 2.2.4. Working Memory

Children’s working memory was assessed using the Digit Span Backwards task, adapted from the working memory subtest of the Wechsler Intelligence Scale for Children–Fourth Edition (WISC-IV) (Zhang 2009). Children were instructed to first listen to a sequence of digits and then repeat them in the reverse order. The sequences were presented through a prerecorded audio by the experimenter, with approximately a 1-s interval between each digit. The sequence length ranged from two to nine with increasing difficulty. Each difficulty level comprised two trials. The length of the longest sequence correctly recalled was recorded for each child. Cronbach’s alpha reliability in our sample was 0.81.

#### 2.2.5. Inhibition

The Expressive Attention task, adopted from the Das–Naglieri Cognitive Assessment System-2 (Naglieri et al. 2014), was used to assess children’s inhibition. Children were presented with three sets of stimuli on separate pages. On the first page, they were instructed to read aloud a set of words (e.g., 红for Red, 蓝for Blue, 黄for Yellow) arranged in an eight-row by five-column grid. On the second page, they were asked to name the color of a grid of color patches (also arranged in eight rows and five columns) of the aforementioned colors. On the third page, children were asked to name the ink color of the printed color words (e.g., the word “Red” might be printed in blue ink) rather than read the word itself, as quickly and accurately as possible. Before each timed trial, participants were given a practice page to ensure they understood the instructions. The response time (in seconds) on the third page was recorded for each child. Cronbach’s alpha reliability in our sample was 0.86.

### 2.3. Procedure

The testing was conducted during regular school hours in various quiet classrooms by four experienced research assistants and lasted about 45 min. The TONI-2 test was initially administered via a computer to confirm the participants in the gifted and matched control groups. No incentives or monetary rewards were given to the participants.

### 2.4. Statistical Analysis

We conducted one-way ANOVAs on the four planning measures to determine if there were any significant differences between our gifted and chronological-age control group. Furthermore, to examine possible mechanisms underlying the group differences, we conducted ANCOVAs with processing speed, working memory, or attention as covariates, separately. The effect sizes for the differences between groups in ANOVAs and ANCOVAs were also calculated, using Cohen’s *d*. Finally, we calculated the correlations between all measures used in this study for the combined sample. All analyses were conducted using IBM SPSS (Statistical Package of the Social Science) software, version 23.0 (IBM Corp 2015).

## 3. Results

The descriptive statistics of the measures we used in our study are presented in Table 1.

Results of ANOVA showed that there was a significant effect of group in CTC total number correct (*F* (1, 72) = 10.97, *p* = 0.001, *d* = 0.77), CTC first move time (*F* (1, 71) = 5.91, *p* = 0.018, *d* = 0.57), and Planned Connections (*F* (1, 72) = 5.72, *p* = 0.019, *d* = 0.56). Compared to controls, gifted children were more accurate in CTC, had longer first move times, and required less time to complete the Planned Connections test. No group differences were found in Planned Search (*F* (1, 72) = 0.70, *p* = 0.407, *d* = 0.19) or Planned Codes (*F* (1, 72) = 0.83, *p* = 0.367, *d* = 0.21). Additionally, gifted children performed better than their controls on Visual Matching (*F* (1, 72) = 6.47, *p* = 0.013, *d* = 0.59).

Next, we performed separate ANCOVAs to examine what factors may account for the significant group differences observed in both the CTC and the Planned Connections tests. Results showed that the significant group differences in CTC total number correct (all *ps* < 0.003) and CTC first move time (all *ps* < 0.034) remained significant even after controlling for processing speed, working memory, or inhibition. In terms of Planned Connections, after controlling for processing speed (as measured by Visual Matching), the group differences were no longer significant (*F* (1, 71) = 1.94, *p* = 0.168, *d* = 0.33). The group differences remained significant after controlling for the effects of inhibition (*F* (1, 71) = 4.00, *p* = 0.049, *d* = 0.47) or working memory (*F* (1, 71) = 4.33, *p* = 0.041, *d* = 0.51).

The *Pearson* correlation coefficients *r* among the variables are presented in Table 2.

The results showed first significant correlations between the three measures of operational planning (*r*s ranged from 0.29 to 0.42). Second, none of the operational planning tasks correlated significantly with CTC (either total number correct or first move time). The CTC scores did not correlate significantly with any of the cognitive measures either. Finally, Visual Matching correlated significantly with all measures of operational planning, and more strongly with Planned Connections.

## 4. Discussion

The present study aimed to examine whether intellectually gifted children perform better than controls in different levels of planning, and whether processing speed, working memory, or inhibition explains these group differences. In contrast to our first hypothesis, we found significant group differences not only in CTC (an action planning task) but also in Planned Connections (an operational planning task). In terms of CTC, although gifted children were more accurate than their chronological-age controls, they were slower in their first move time. This suggests that gifted children may spend more time creating a representation of the problem and going through different scenarios on how to solve the problem before they make their first move. In terms of Planned Connections, the observed differences may be explained by the speed requirements of the task, since the significant differences became nonsignificant after controlling for speed of processing. According to Demetriou et al. (2014), speed is a significant predictor of cycle transitions in the development of fluid intelligence (including the age group of our participants) that also includes measures of problem solving. However, the significant group differences in Planned Connections may also relate to the fact that in some of the Planned Connections items, children had to shift between numbers and letters, and we know that gifted children have better cognitive flexibility (i.e., shifting) than chronological-age controls. Similar to Montoya-Arenas et al. (2018), Rocha et al. (2020), and Vogelaar et al. (2019), we found no significant differences between groups in the other two measures of operational planning. Taken together, our findings suggest that differences between gifted and controls can primarily be observed in tasks that require higher-level cognitive planning (Das and Misra 2015). It is important to note here that neither the CTC total number correct nor the CTC first move time correlated significantly with any of the operational planning tasks (see Georgiou et al. 2017, for a similar finding). This was expected to some degree because they are representing different levels in the hierarchy of planning in the activity theory (Leontjev 1978). However, it is also possible that individual differences in higher-level planning measures, such as CTC, are influenced by processes that are different from those involved in rudimentary planning tasks.

In regard to our second goal, we found that the group differences in CTC accuracy and first move time remained significant even after controlling for the effects of processing speed, working memory, or inhibition. In view of the significant group differences in processing speed in favor of the gifted children (see Table 1) and the significantly longer CTC first move time by the gifted children, our finding suggests that processing speed is not playing a key role in action planning (at least when action planning is operationalized with CTC). Interestingly, CTC did not correlate significantly with any of the other cognitive tasks either. There might be two explanations for this finding. First, some researchers have argued that Digit Span Backwards might be rather shallow in its processing demands (e.g., Colom et al. 2005; St Clair-Thompson 2010), taxing more storage than processing. To the extent this is true, it may explain the nonsignificant correlations with CTC because CTC relies heavily on the processing of information as it becomes available. It also worth noting here that we found no significant group differences in Digit Span Backwards (see Table 1), which is in contrast to the finding of previous studies with gifted children that used more complex working memory tasks such as the reading span task (e.g., Aubry et al. 2021, see also Rodríguez-Naveiras et al. 2019, for a discussion on this). Second, because our CTC task only included two to four lines of information, and each line contained three to four colored chips, it is possible that children did not have to inhibit a lot of competing information in order to find an answer to the problem. In contrast to CTC, significant group differences in Planned Connections became non-significant after controlling for processing speed. Again, this highlights the need to consider the level that different measures of planning represent, as depending on the level of planning included in a given study, one may or may not detect significant differences between gifted children and their chronological-age controls.

Some limitations of the present study are worth mentioning. First, due to time restrictions, we were only able to administer one action planning task. Future studies should replicate our findings with more measures of action planning. Second, for the same reason mentioned above, we only administered a single measure of processing speed, working memory, and attention. This may have weakened our constructs and reduced our chances of finding significant effects when we used these constructs as covariates. We also acknowledge here that our attention task (Expressive Attention from CAS-2) measures only inhibition. It is possible that it is the ability to sustain attention for a period of time (i.e., CTC requires more time to finish each item than any of the items in Planned Search, Planned Connections, or Planned Codes) and not inhibition per se that matters in action planning tasks. A future study should also include measures assessing orienting and alertness. Third, we did not administer any measures of activity planning. By definition, activity planning involves long-term scheduling and deployment of strategies. This would require following up participants over an extended period of time to see whether and how they met their long-term goals. Unfortunately, this was not an option for us because the schools had only given us a certain amount of time to complete our testing. Fourth, as indicated in the Method section, our choice of planning tasks was to a large extent dictated by the focus of the special issue on PASS processes. Clearly, our findings need to be replicated with a broader range of planning and cognitive tasks. Finally, our sample was recruited from an underdeveloped rural area in Anhui province, and, as a result, our findings may not generalize to children from metropolitan cities in China.

To conclude, our findings add to a growing body of studies examining the role of planning in gifted children (e.g., Bucaille et al. 2023; Georgiou et al. 2022; Montoya-Arenas et al. 2018; Vogelaar et al. 2019) by showing that we need to consider an important parameter in the conversation around planning, namely, that of the level of planning. As shown here, we found persistent differences between the gifted and controls only in CTC, a measure of action planning. We found no significant group differences in two of the measures of operational planning. This suggests that despite Best et al.’s (2011) characterization of the CAS Planning task as indicators of “complex EF”, they are still capturing lower-level planning that may not be adequate to differentiate gifted children from their chronological-age controls. An obvious practical implication of our findings is that if planning tasks are used for the selection of gifted individuals (see Naglieri and Kaufman’s 2001 argument), we need to assess children in action planning rather than operational planning.

## Figures and Tables

**Table 1:** Performance of Gifted Children and Controls on Different Measures.

Variables	Group	Mean	*SD*	Min	Max	*F*	*p*	Cohen’s *d*
Nonverbal IQ ^a^	Gifted	98.09	0.89	97.00	99.00	171.77	0.001	3.22
	Controls	65.36	14.74	42.00	88.00			
CTC total number correct	Gifted	6.37	2.46	2.00	9.00	10.97	0.001	0.77
	Controls	4.44	2.55	0.00	9.00			
CTC first move time	Gifted	17.69	13.00	1.00	60.14	5.91	0.018	0.57
	Controls	11.44	8.71	2.00	38.01			
Planned Connections	Gifted	190.14	47.41	106.00	287.00	5.72	0.019	0.56
	Controls	224.51	72.19	117.00	448.06			
Planned Codes	Gifted	146.86	29.56	72.00	205.00	0.83	0.367	0.21
	Controls	141.10	24.92	81.00	181.00			
Planned Search	Gifted	5.77	1.53	3.60	10.40	0.70	0.407	0.19
	Controls	5.54	0.79	4.30	7.20			
Visual Matching	Gifted	47.95	4.62	34.06	59.00	6.47	0.013	0.59
	Controls	45.33	4.21	37.00	53.00			
Digit Span Backwards	Gifted	6.94	1.78	2.00	9.00	1.24	0.269	0.24
	Controls	6.51	1.54	2.00	9.00			
Expressive Attention	Gifted	45.03	11.20	28.00	67.00	2.87	0.095	0.39
	Controls	49.49	11.39	31.00	78.00			

*Note.* ^a^ Percentile. CTC = Crack the Code. The denominator degrees of freedom for the *F* values were 72 for all planning measures, except for CTC first move time, which was 71 due to one child failing to start the formal test.

**Table 2:** *Pearson* Correlations Among Study Variables for Gifted Children and Controls Combined.

Variables	1.	2.	3.	4.	5.	6.	7.	8.
1. CTC total number correct	-							
2. CTC first move time	0.530 **	-						
3. Planned Connections	−0.148	−0.115	-					
4. Planned Codes	−0.115	0.010	−0.359 **	-				
5. Planned Search	−0.029	0.124	0.416 **	−0.288 *	-			
6. Visual Matching	0.120	0.181	−0.463 **	0.338 **	−0.323 **	-		
7. Digit Span Backwards	0.152	0.135	−0.238 *	−0.085	−0.206	0.087	-	
8. Expressive Attention	−0.122	−0.108	0.273 *	−0.155	0.020	−0.247 *	−0.160	-

*Note. n* = 74; * *p* < .05, ** *p* < .01.

## Data Availability

The data presented in this study are available upon request from the corresponding authors due to ongoing work using the same database.
